# UBTF facilitates melanoma progression via modulating MEK1/2-ERK1/2 signalling pathways by promoting GIT1 transcription

**DOI:** 10.1186/s12935-021-02237-8

**Published:** 2021-10-18

**Authors:** Jian Zhang, Jiaojiao Zhang, Wenli Liu, Rui Ge, Tianyuan Gao, Qiong Tian, Xin Mu, Lingyu Zhao, Xu Li

**Affiliations:** 1 Department of Dermatology, The First Affiliated Hospital of Xi’an Jiaotong University, Xi’an, 710061 China; 2 Department of Dermatology, The Third Hospital of Yulin, Yulin, 719000 Shaanxi China; 3 Department of Cell Biology and Genetics, Institute of Genetics and Developmental Biology, School of Basic Medical Sciences, Xi’an Jiaotong University Health Science Center, Xi’an, 710061 China; 4 Center for Translational Medicine, The First Affiliated Hospital of Xi’an Jiaotong University, Xi’an, 710061 China; 5 Key Laboratory for Tumor Precision Medicine of Shaanxi Province, The First Affiliated Hospital of Xi’an Jiaotong University, Xi’an, 710061 China

**Keywords:** UBTF, GIT1, Melanoma, Proliferation, MEK1/2-ERK1/2 signalling pathways

## Abstract

**Background:**

UBTF is an HMGB-box DNA binding protein and a necessary Pol I/Pol II basal transcription factor. It has been found that UBTF involves in carcinogenesis and progression of a few cancers. Nevertheless, the the biological function and potential molecular mechanism of UBTF in melanoma are still not clear and need to be clarified.

**Methods:**

UBTF and GIT1 expressions in melanoma specimens and cell lines were examined by quantitative real-time PCR (qRT-PCR) and Western blot. MTT and colony formation assays were used to investigate the effects of UBTF and GIT1 on melanoma cell proliferation. Cell cycle and apoptosis assays were detected by flow cytometry. Tumor formation assay was used to analyze the effect of UBTF on melanoma growth. Bioinformatics predicting, chromatin immunoprecipitation (ChIP)-qRT-PCR and reporter gene assay were fulfilled for verifing GIT1 as UBTF targeting gene.

**Results:**

Here we reported that UBTF mRNA and protein expressions were upregulated in primary melanoma specimens and cell lines. UBTF overexpression facilitated melanoma cell proliferation and cell cycle progression and restrained. Silencing UBTF suppressed cell multiplication, cell cycle progression and tumor growth, and promoted apoptosis. UBTF expression was positively related with GIT1 expression in human melanoma tissues. It was verified that UBTF promoted GIT1 transcription in melanoma cells through binding to the promoter region of GIT1. Furthermore, GIT1 overexpression promoted melanoma cell growth and suppressed apoptosis. Knockdown of GIT1 inhibited cell multiplication and induced apoptosis. Overexpression of GIT1 eliminated the effects of silencing UBTF on melanoma cells. Importantly, UBTF activated MEK1/2-ERK1/2 signalling pathways by upregulating GIT1 expression.

**Conclusions:**

Our study demonstrates that UBTF promotes melanoma cell proliferation and cell cycle progression by promoting GIT1 transcription, thereby activating MEK1/2-ERK1/2 signalling pathways. The findings indicate that UBTF plays a crucial function in melanoma and may be a potential therapeutic target for the treatment of this disease.

**Supplementary Information:**

The online version contains supplementary material available at 10.1186/s12935-021-02237-8.

## Background

Melanoma, a prevalent lethal malignancy, is one of the most aggressive and intractable cancers. In recent years, melanoma morbidity keeps increasing in the world [1, 2]. The melanoma morbidity is remarkable difference among different populations, with high morbidity in Caucasian and low morbidity in Asian and African [3]. On the basis of the statistics, there are still 91,270 new melanoma patients and 9320 deaths in the United States [4]. The statistics data of China reveals that there are 8000 new sufferers and 3200 deaths [5]. In China, the melanoma morbidity is lower, however, the mortality is observably higher. The therapy of melanoma contains surgery, radiotherapy, chemotherapy, gene therapy and immunotherapy. Although significance advances in melanoma therapy have been acquired, the therapeutic effect remains still dissatisfied. The 5-year survival rate of sufferers is only 30–40% [6]. The ultimate causes of the unsatisfactory are insufficient on molecular mechanism of melanoma oncogenesis and development. Hence, it is imminently needed to confirm the molecular pathogenesis of melanoma for achieving better therapeutic effect.

Upstream binding transcription factor (UBTF) is a multiple HMGbox architectural protein. It is found that Pol I transcription factor UBTF has a double function in the modulation of Pol I and Pol II mediated transcription. UBTF is not only considered to play a role in RNA Pol I-specific transcription of the ribosomal genes, but also enrich at Pol II-transcribed genes throughout human genomes [7, 8]. UBTF participates in DNA damage and repair containing mediators of ATR/ATM-regulated DNA damage response [8]. UBTF also involves in the responds of growth factor stimulation. UBTF can regulate differentiation, proliferation, and cell growth through different signalling pathways [9]. The role of UBTF is damaged via reciprocity with repressive proteins of cell multiplication, such as Rb, p130 and p53 [10]. The function of UBTF in cancers has not been extensively studied. It was reported that UBTF expression upregulated in lung cancer specimens [11]. Recently, studies found that UBTF involved in oncogenesis and development in a few tumor [12, 13]. Nevertheless, the function of UBTF in many kinds of tumors, including melanoma, hasn’t been investigated exactly. Especially, the potential mechanisms underlying the role of UBTF are still not clear.

In this study, we detected the UBTF expression level in 66 melanoma sufferers, and explored the function and corresponding mechanisms of UBTF in modulating melanoma progression. The data showed that UBTF expression was observably increased in melanoma and the high UBTF expression was associated with clinical pathological characteristics. UBTF facilitated melanoma cell proliferation and suppressed apoptosis. Through chromatin immunoprecipitation-qRT-PCR (ChIP-qRT-PCR) and reporter gene assay, we further confirmed that UBTF promoted melanoma cell growth and cell cycle progression by facilitating G-protein-coupled receptor kinase-interacting protein 1 (GIT1) transcription, thereby activating MEK1/2-ERK1/2 signalling pathways.

## Methods

### Melanoma clinical specimens

Human melanoma and adjacent non-tumor tissues were gathered from 66 sufferers at the Department of Pathology, the First Affiliated Hospital of Medical College, Xi’an Jiaotong University, PR China. These sufferers hadn’t been treated before surgery. According to the sufferers’ pathology records, clinical pathological features such as age, sex, anatomic site, tumor thickness, lymph node metastasis and initial stage were collected. Before collection of specimens, the informed consents were acquired from each sufferer. This research was authorized by the Ethical Committee of the First Affiliated Hospital of Medical College, Xi’an Jiaotong University.

### Animals

Male BALB/c nude mice (4 week-old) were purchased from Yangzhou University and were fed under aseptic condition. The researchers were blinded to the group allocation in animal experiment. Institutional Animal Care and Use Committee of the First Affiliated Hospital of Medical College, Xi’an Jiaotong University authorized these animal experiments. On the basis of institution’s guidelines of laboratory animals, we fulfilled animal experiments.

### Cell culture

Melanoma cell lines (Mel-RM, A375 and SK-MEL-28) and human keratinocyte HaCaT cell were bought from the Cell Bank (Shanghai institutes for Biological Sciences). These cell lines have been identificated and detected by the Cell Bank. Cells were cultivated in Dulbecco’s Modified Eagle’s Medium (DMEM, Gibco, MD, USA), supplemented with 10% fetal calf serum (Gibco), and incubated in 5% CO_2_ incubator at 37 °C.

### siRNA synthesis and transfection

Small interfering RNAs (siRNAs) were designed and synthesized for silencing UBTF and GIT1 expressions by GenePharma (SGC, Shanghai, China). These sequences of siRNA were showed in Additional file 4: Table S1. After cultivating A375/SK-MEL-28 cells for 24 h, UBTF siRNAs and GIT1 siRNAs were instantaneously transfected into melanoma cells with Jet Prime (Polyplus-transfection, Ilkirch, France) according to the manufacturer’s protocol.

### Plasmids and transfection

The plasmids were constructed by inserting the full length of UBTF and GIT1 into pCMV2-GV146 vector (Genechem Co. Ltd, China), respectively. The reporter plasmid pGL3-GIT1 containing a 424-bp fragment spanning from chr17: 27915862 to 27916285 relative to the GIT1 promoter, put upstream of the firefly luciferase reporter gene (pGL3-GIT1-luc, Genechem Co. Ltd, China). A375/SK-MEL-28 cells were cultivated in DMEM medium for 24 h. Next, pCMV2-GV146 vector, pCMV2-GV146-UBTF vector, or pCMV2-GV146-GIT1 vector were transiently transfected into melanoma cells with Jet Prime (Ilkirch) for following experiment.

### Lentiviral construction and transfection

UBTF shRNA and control were inserted into the lentiviral vectors (Genechem Company Ltd.), UBTF shRNA lentiviral vector was constructed for silencing UBTF. These sequences were showed in Additional file 4: Table S2. Lentiviral vector including invalid short hairpin RNA was known as negative control (sh-Ctrl). A375 cells were planted in 12-well plates and infected by using 0.5 ml viral stock for 10 h. Next, the solution was substituted with DMEM supplemented with fetal calf serum.

### MTT assay

A375/SK-MEL-28 cells were planted at 3000 cells/well in 96-well plates. Cell activity was detected through performing MTT assay (Sigma, MO, USA) after treatment. After the intervention, these cells was added by 20 µl MTT diluent/well and cultured for 4 h in incubator. Next, suspension liquid was thrown away and 150 µl dimethylsulfoxide (Sigma Louis, USA) was used to dissolve formazan crystals. The assay value was determined through using multi-microplate test system (CLARIOstar, BMG LABTECH, USA).

### Colony formation assay

A375/SK-MEL-28 cells were concentrated in 12-well plates in the number of 500 per well 24 h after intervention. Then, these cells were cultivated with ordinary medium for 11 days. Cell colonies were fixed by Methanol for 10 min, stained with 0.2% crystal violet for 30 min. Four parallel wells were performed for each group.

### Cell cycle assay

Melanoma cells were planted in 12-well plates and intervented for 24 h. A375/SK-MEL-28 cells were washed, digested, and resuspended in phosphate-buffered saline (PBS). Then, ice-cold ethanol (70%) was applied to immobilize these treated cells at 4 °C overnight. The cells were gathered as single cell suspensions, washed twice with PBS, and resuspended in PBS. RNase A (0.1 mg/ml) and propidiumiodide (0.05 mg/ml) were appended to these cells and incubated for 20 min. The proportion of cell cycle was determined by using flow cytometry (Beckman, Miami, USA).

### Apoptosis assay

A375/SK-MEL-28 cells were intervented for 48 h and harvested. The cells were washed thrice with PBS and resuspended as single cell suspensions. Then, these cells were stained with an Annexin-V-FITC Apoptosis Detection Kit (Sigma-Aldrich, USA) according to the instructions. Apoptosis level was measured and analyzed by using flow cytometry (Beckman, Miami, USA).

### Tumor formation assay

A375 cells were infected with sh-Ctrl or UBTF shRNA and were then resuspended with DMEM. Male 5-week-old BALB/c nude mice were applie to measure tumor formation. Institutional Committee of Animal Use at Xi’an Jiaotong University authorized this animal experiment. The infected A375 cells (2 × 10^6^) inoculated subcutaneously into both posterior flanks of the mice. Tumor volume was measured through using vernier caliper every 3 d after injection. At 31 days, the nude mice were anesthetized through inhalation of 3% isofluorane and given one subcutaneous dose of carprofen (8 mg/kg) for euthanasia. The length (L) and width (W) of tumor were applied to figure up the volume (V). Formula: V = (L ×W^2^)/2. The tumors were stripped and detected weight 31 d after injection. These tumor specimens were frozen storage for following experiment.

### Chromatin immunoprecipitation (ChIP)-qRT-PCR and -RT-PCR

According to previous research methods [14], we fulfilled ChIP assay. Formaldehyde (1%) was used to crosslink for fifteen min in A375/SK-MEL-28 cells and glycine was applied to quench. Ultrasonic processor was used to sonicate melanoma cells and extract nuclear lysates. The chromatin was broken into approximately 200 bp DNA fragments with ultrasonic processing. These DNA fragments were incubated through using UBTF or IgG antibodies for 12 h 4 °C, respectively (Additional file 4: Table S3). Then, Dynabeads Protein A (Thermo Fisher Scientific, USA) was applied to obtain DNA-protein complexes and TE buffer was used to elute the complexes at 65 °C. Next, these complexes were treated for reversing crosslinking 8 h at 65 °C. DNA was purified with the QIA quick PCR purification kit (QIAGEN, Germany). These DNA products were verified by applying qRT-PCR and RT-PCR. The correlative gene-specific primers were revealed in Additional file 4: Table S4.

### Reporter gene assay

A375/SK-MEL-28 cells were cultivated in 96-well plates. There were five parallel wells each group. Melanoma cells were transfected with pGL3-luc or pGL3-GIT1-luc, and were co-transfected with pGL3-GIT1-luc and UBTF siRNAs or GIT1 overexpression vector for 48 h. Trypan blue was used to stain these cells for detecting the luciferase activity. Next, we examined and analyzed the luciferase activity through applying the Dual-Luciferase Reporter System (Promega, USA).

### Quantitative RT-PCR (qRT-PCR)

TRIzol was applied to extract RNA from melanoma specimens or cell lines on the basis of specified methods (Invitrogen, CA, USA). These RNA specimens were examined with Nanodrop 2000 (Thermo Scientific, USA) and the OD260/OD280 specific value ranging from 1.9 to 2.0 were regarded as good quality. cDNA was reversed transcription in the light of the specification (Takara, Dalian, China). Quantitative polymerase chain reaction (qRT-PCR) assay was carried out with the SYBR Green qRT-PCR kit (Applied Biosystems, CA, USA). These correlative primers were showed in Additional file 4: Table S4. The qRT-PCR reactions were fulfilled by applying the IQ5 Multicolor qRT-PCR Detection System (Bio-Rad, USA). GAPDH was regarded as internal control for gene mRNAs.

### Western blot

Melanoma specimens or cell lines were use to extract protein through applying RIPA buffer (KenGEN, China). The protein was quantified with BCA Protein Assay Kit (Beyotime, China). Equal amounts of protein solution were separated in 10% SDS-PAGE gels followed by transfer onto PVDF membranes. Nonfat milk (5%) was used to block the membranes. Next, these membranes were blotted with relevant primary antibodies for 12 h at 4 °C. These primary antibodies were showed in Additional file 4: Table S3. After that, these membranes were incubated by using the homologous secondary antibodies for 2 h. Proteins were measured through using ECL (GE Healthcare, Beijing, China). Chemiluminescence examination was performed with Syngene GBox (Syngene, UK).

### Statistical analysis

SPSS24.0 software was applied to analyze the data. Mean ± SD represented the data. All data came from at least three independent trials. The data was analyzed by Student’s t-test or one-way ANOVA. Pearson’s correlation analysis was used to evaluate the association between UBTF and GIT1. The relationships between UBTF or GIT1 and clinical pathological features were evaluated by Chi-square test. Value of p < 0.05 is statistically significant.

## Results

### UBTF is evidently upregulated in melanoma and is related to clinicopathologic characteristics

**Fig. 1:**
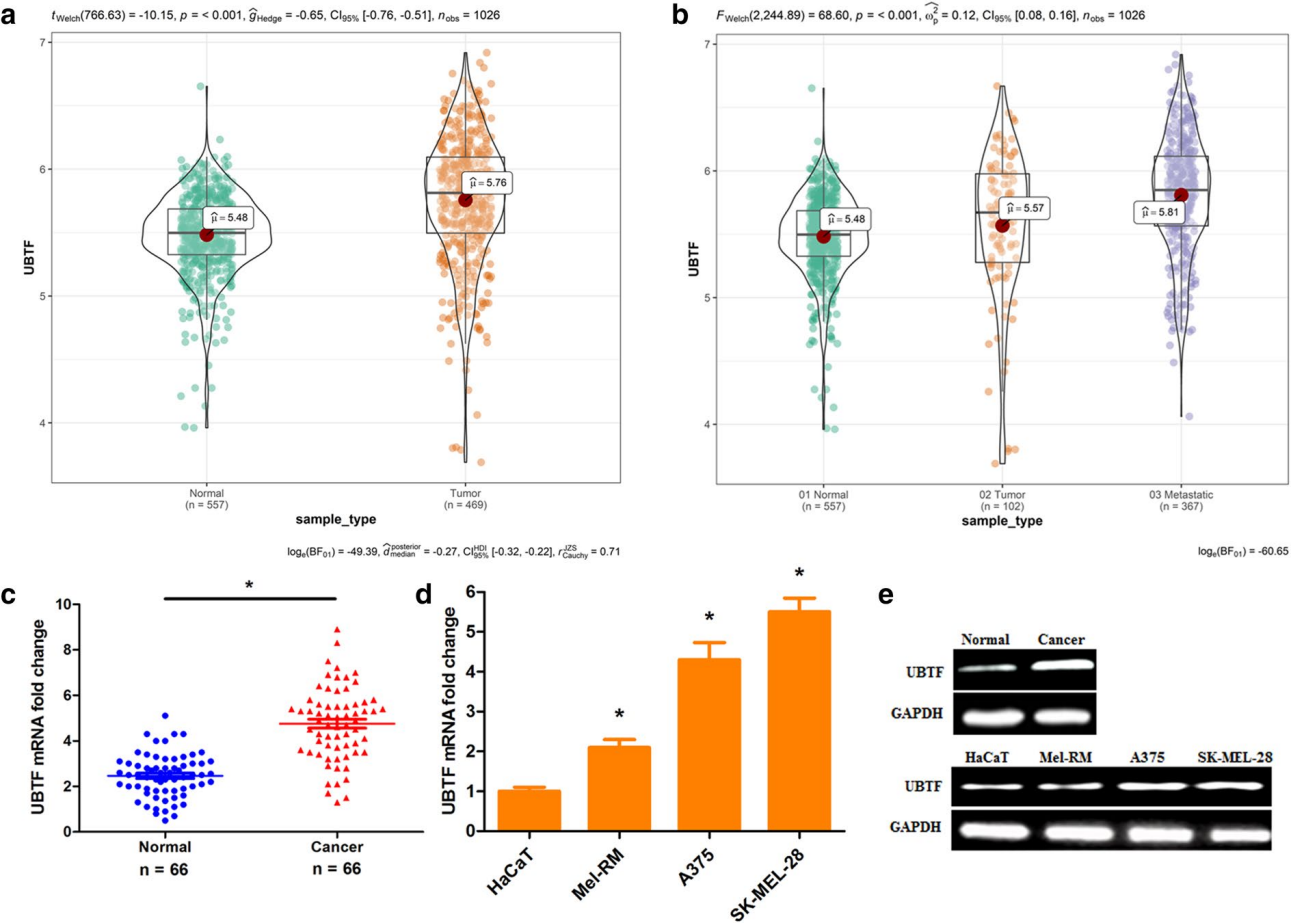
UBTF expression upregulates in human melanoma samples and cell lines. **a** TCGA data showed that UBTF expression was increased in melanoma. **b** TCGA data revealed that UBTF expression was high in metastatic melanoma tissues. **c** UBTF mRNA expression was significantly increased in melanoma samples compared with normal skin samples. **d** UBTF mRNA expression was observably enhanced in melanoma cell lines (Mel-RM, A375 and SK-MEL-28) compared with human keratinocyte HaCaT cells. **e** UBTF protein expression was upregulated in melanoma tissues and cell lines. *p < 0.01

To investigate the function of UBTF in melanoma progression, we analyzed and detected UBTF expression in melanoma specimens and cell lines. The Cancer Genome Atlas (TCGA) data revealed that the UBTF expression was observably upregulated in melanoma samples compared with normal skin tissues (Fig. 1a; p < 0.001). The UBTF expression was also remarkable higher in metastatic melanoma tissues than in tumor and normal skin tissues (Fig. 1b; p < 0.001). The qRT-PCR results showed that UBTF mRNA level was significantly increased in 80.3% (53/66) of the melanoma tissues compared to the normal tissues (Fig. 1c; p < 0.001). The UBTF mRNA expression was also markedly upregulated in melanoma cell lines (Mel-RM, A375 and SK-MEL-28) than in human keratinocyte HaCaT cells (Fig. 1d; p < 0.001). The UBTF protein expression was significantly upregulated in melanoma tissues and cell lines (Fig. 1e). Moreover, the high UBTF expression was associated with melanoma thickness, lymph node metastasis and initial stage. Nevertheless, the expression was not related to age, gender and anatomic site (Additional file 4: Table S5). The results indicated that UBTF might be involved in human melanoma progression.

### UBTF facilitates melanoma cell growth in vitro and in vivo

**Fig. 2:**
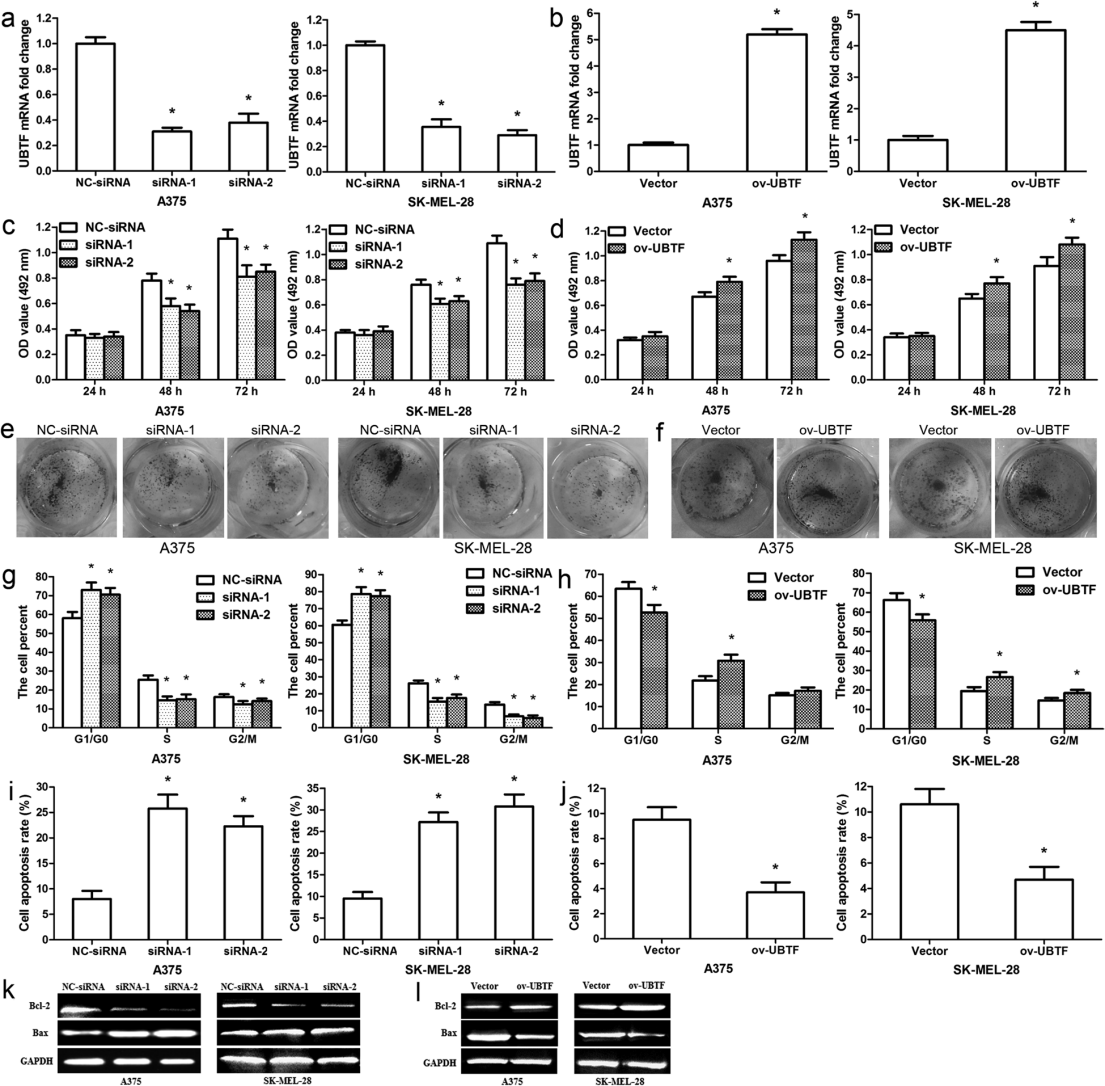
UBTF promotes melanoma cell proliferation and restrains apoptosis in vitro. **a** UBTF mRNA level was measured in A375/SK-MEL-28 cells after transfection with UBTF siRNAs. **b** UBTF mRNA level was detected after UBTF overexpression. **c** MTT assay revealed melanoma cell activity after transfection with UBTF siRNAs. **d** MTT assay showed melanoma cell activity after transfection with UBTF overexpression vector. **e** Cell colonies were measured 11 days after transfection with UBTF siRNAs. **f** Cell colonies were detected 11 days after transfection with UBTF overexpression vector. **g** Cell cycle was analyzed by using flow cytometry. The histograms revealed the proportion of G1/G0, S and G2/M phase cells after transfection with UBTF siRNAs. **h** The histograms showed the percentage of G1/G0, S and G2/M phase cells after transfection with UBTF overexpression vector. **i** The data represented the percentage of apoptosis after transfection with UBTF siRNAs. **j** The data revealed the ratios of apoptosis after transfection with UBTF overexpression vector. **k** Bcl-2/Bax expressions were detected after transfection with UBTF siRNAs. **l** Bcl-2/Bax expressions were measured after transfection with UBTF overexpression vector. *p < 0.01, n = 3

To explore the function of UBTF in human melanoma, A375/SK-MEL-28 cells were transfected with UBTF siRNAs, negative control, UBTF overexpression vector, or control empty vector, independently. qRT-PCR results showed that UBTF siRNAs remarkably decreased UBTF mRNA expression in A375/SK-MEL-28 cells (Fig. 2a; p < 0.01), and UBTF overexpression vector evidently enhanced UBTF mRNA expression (Fig. 2b; p < 0.01). Results from MTT assays revealed that silencing UBTF significantly restrained melanoma cell proliferation at 48 and 72 h after transfection (Fig. 2c; p < 0.01), whereas UBTF overexpression observably promoted melanoma cell multiplication (Fig. 2d; p < 0.01). Colony formation assays also showed the similar results (Fig. 2e, f). Cell cycle analysis discovered that silencing UBTF led to the accumulation of cells at the G1/G0 phase and reduced the ratio of S and G2/M phase (Fig. 2g; p < 0.01); nevertheless, UBTF overexpression remarkably decreased the proportion of G0/G1 phase cells and enhanced the the proportion of S and G2/M phase cells (Fig. 2h; p < 0.01). In addition, analysis of cell apoptosis confirmed that silencing UBTF clearly increased the proportion of apoptosis cells, while UBTF overexpression observably reduced the proportion of apoptosis (Fig. 2i, j; p < 0.01). It was also found that silencing UBTF downregulated Bcl-2 protein expression and upregulated Bax protein expression in melanoma cell (Fig. 2k), whereas UBTF overexpression increased Bcl-2 expression and decreased Bax expression (Fig. 2l).

**Fig. 3:**
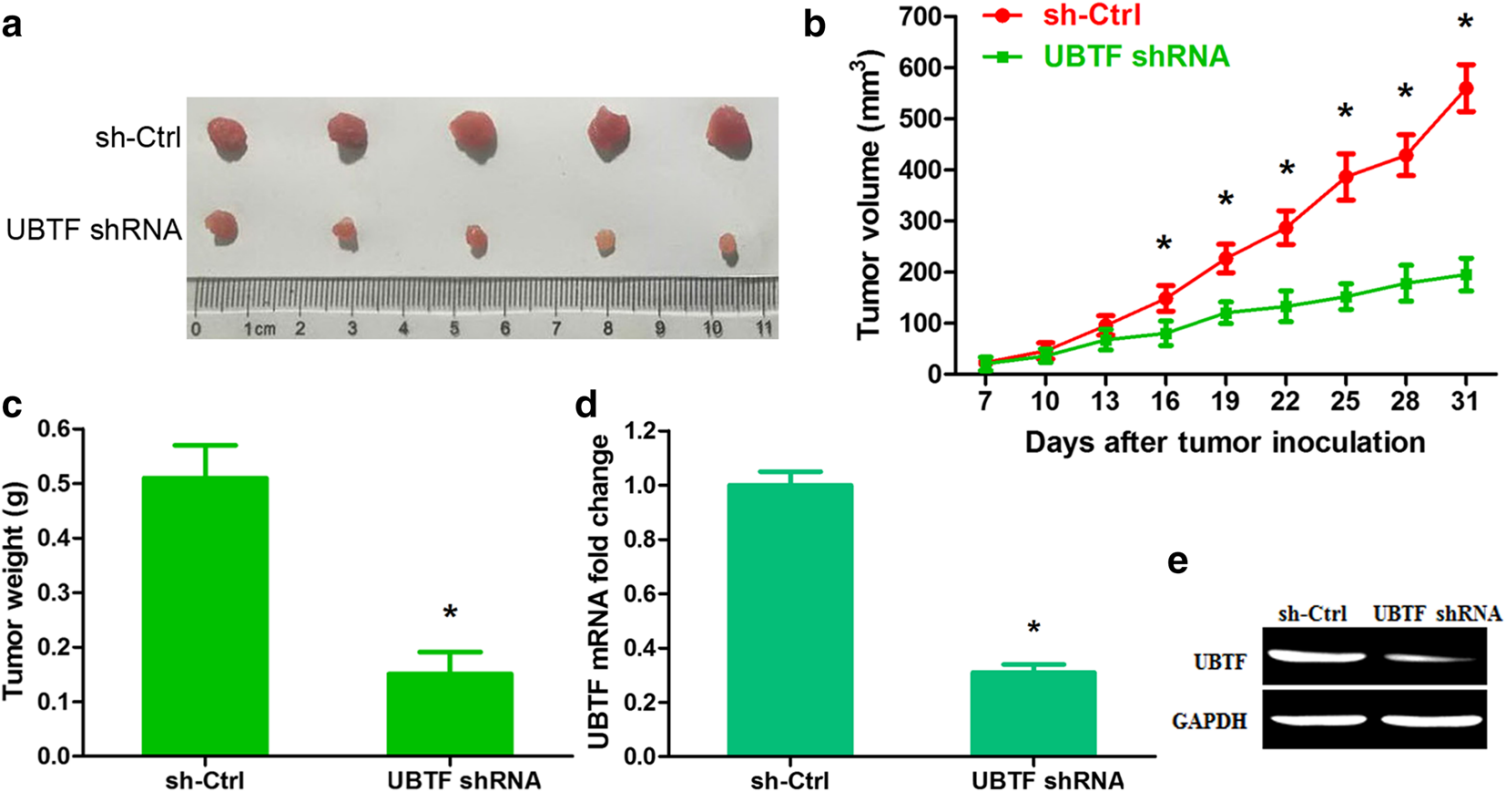
UBTF accelerates melanoma cell multiplication in vivo. **a** Morphology of isolated tumors from nude mice. **b** Growth curvatures of tumor volume were obtained from 7 to 31 days. **c** Tumors were weighed at 31 day after initial injection. **d** UBTF mRNA expression in xenograft tumor was measured by qRT-PCR. **e** UBTF protein expression in xenograft tumor was detected by Western blot. *p < 0.01, n = 5

To further detect the role of UBTF in melanoma progression in vivo, artificial UBTF shRNA lentiviral vector was constructed and obtained a steady A375 cell clone. UBTF expression was significantly decreased at both mRNA and protein levels in A375 cell (Additional file 1: Fig. S1a, b; p < 0.01). UBTF shRNA remarkably suppressed melanoma cell growth, as evidenced by MTT and colony formation assays (Additional file 1: Fig. S1c, d; p < 0.01). UBTF shRNA resulted in an increase in cells of G1 phase and a decrease in cells of S and G2/M phases (Additional file 1: Fig. S1e; p < 0.01). UBTF shRNA also induced apoptosis (Additional file 1: Fig. S1f; p < 0.01). Next, sh-Ctrl-infected and UBTF shRNA-infected A375 cells were injected subcutaneously and tumor growth was observed. The data of tumor size and weight revealed that tumor growth was evidently restrained by UBTF shRNA as compared with that seen in sh-Ctrl group (Fig. 3a–c). At day 31, the mean volume of tumors with sh-Ctrl was about 2.87-fold higher than that of tumors with UBTF shRNA (Fig. 3b; n = 5, p < 0.01). The mean weight of tumors was 0.51 g in sh-Ctrl group and 0.15 g in UBTF shRNA group (Fig. 3c; p < 0.01). The downregulation of UBTF in tumors derived from UBTF shRNA-transduced A375 cells was verified at mRNA and protein expressions (Fig. 3d, e; p < 0.01). These findings demonstrated that UBTF facilitated human melanoma cell proliferation and suppressed apoptosis. Nevertheless, many factors and molecular networks participate in the occurrence and development of melanoma. Hence, we further research the molecular mechanism of UBTF modulation in human melanoma.

### UBTF accelerates GIT1 gene transcription through binding to its promoter

**Fig. 4:**
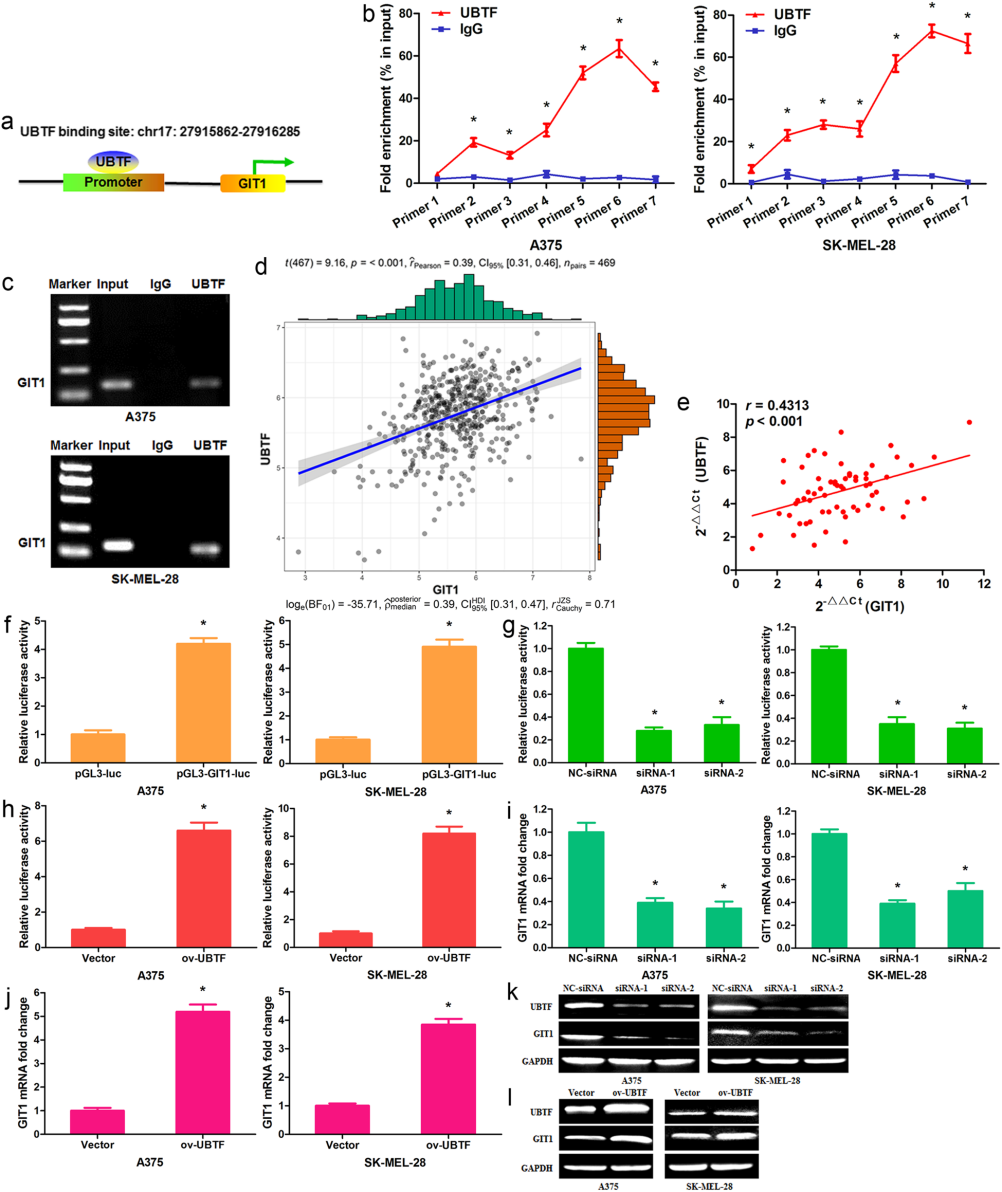
UBTF binds the promoter region of GIT1 in melanoma cell. **a** Bioinformatics analysis (UCSC Genome Browser) predicted the UBTF binding site in the promoter of GIT1. **b** ChIP-QPCR showed that UBTF bound to the promoter region of GIT1 in A375/SK-MEL-28 cells. **c** ChIP-RT-PCR revealed that UBTF bound to the promoter region (Primer 6) of GIT1. **d** TCGA data showed that UBTF expression was positively correlated with GIT1 expression in human melanoma tissues. **e** qRT-PCR assay revealed the positive correlation between UBTF and GIT1 expressions. **f** A375/SK-MEL-28 cells were transfected with pGL3-GIT1-luc (target sequences of UBTF), the luciferase activity was examined at 48 h after transfection. Renilla luciferase was known as the internal control. **g** A375/SK-MEL-28 cells were co-transfected with pGL3-GIT1-luc and UBTF siRNAs; the luciferase activity was determined. **h** A375/SK-MEL-28 cells were co-transfected with pGL3-GIT1-luc and UBTF overexpression vector; the luciferase activity was measured. **i** UBTF siRNAs reduced GIT1 mRNA expression in melanoma cells. **j** UBTF overexpression vector enhanced GIT1 mRNA expression in melanoma cells. **k** UBTF siRNAs restrained GIT1 protein expression in melanoma cells. **l** UBTF overexpression vector promoted GIT1 protein expression in melanoma cells. *p < 0.01, n = 3

In order to further analyze the molecular mechanism of UBTF regulating melanoma progression, UBTF target gene was predicted by bioinformatics analysis (UCSC Genome Browser). It was found that UBTF could bind to the promoter region of GIT1 (Fig. 4a). Chromatin immunoprecipitation (ChIP)-QPCR assay verified that UBTF bound to the promoter of GIT1 in A375/SK-MEL-28 cells (Fig. 4b). ChIP-RT-PCR assay also confirmed that UBTF bound to the promoter (Primer 6) of GIT1 in melanoma cells (Fig. 4c). TCGA data showed that UBTF expression was positively related with GIT1 expression in human melanoma tissues (Fig. 4d; n = 469, r = 0.39, p < 0.001). In normal tissues, UBTF expression was positively correlated with GIT1 expression (Additional file 2: Fig. S2a; n = 557, r = 0.43, p < 0.001). In all tissues, there was a positive correlation between UBTF expression and GIT1 expression (Additional file 2: Fig. S2b; n = 1026, r = 0.47, p < 0.001). The qRT-PCR results also showed that UBTF mRNA expression was remarkably positively correlated with GIT1 mRNA expression in melanoma (Fig. 4e; n = 66, r = 0.4313, p < 0.001, Pearson’s correlation). The promoter reporter assay was performed to measure if UBTF bound to the promoter of GIT1. The target sequence of promoter of GIT1 was inserted into the luciferase gene in the pGL3 reporter plasmid. Luciferase activity was detected at 48 h post-transfection in A375/SK-MEL-28 cells. The data revealed that luciferase activity evidently increased in pGL3-GIT1-luc group as compared with in pGL3-luc group (Fig. 4f; p < 0.01). After pGL3-GIT1-luc and UBTF siRNAs were co-transfected, luciferase activity remarkably reduced in UBTF siRNA groups as compared with NC-siRNA group (Fig. 4g; p < 0.01). After pGL3-GIT1-luc and UBTF overexpression vector were co-transfected, luciferase activity significantly increased in UBTF overexpression vector group compared with control vector group (Fig. 4h; p < 0.01). Moreover, we analyzed the effect of UBTF on GIT1 expression. It was found that UBTF siRNAs observably suppressed GIT1 mRNA expression in A375/SK-MEL-28 cells, while UBTF overexpression vector significantly enhanced GIT1 mRNA expression (Fig. 4i, j; p < 0.01). UBTF siRNAs decreased UBTF and GIT1 protein levels, UBTF overexpression vector increased UBTF and GIT1 protein levels (Fig. 4k, l). The findings demonstrated UBTF as a transcriptional regulator of GIT1 in human melanoma cells.

### GIT1 is frequently increased in melanoma and relevant to pathological features

**Fig. 5:**
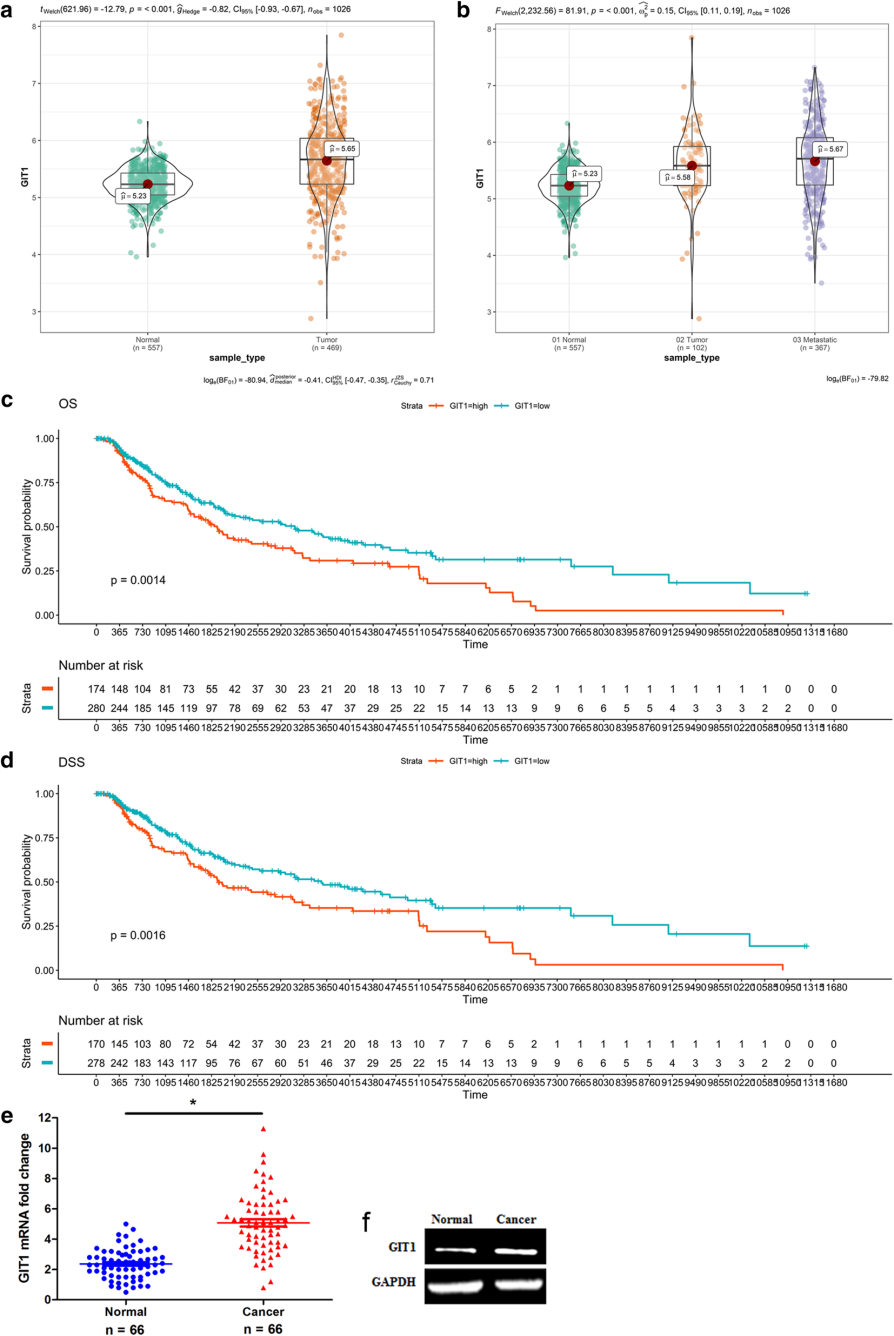
GIT1 expression increases in human melanoma tissues. **a** TCGA data revealed that GIT1 expression was upregulated in melanoma. **b** TCGA data showed that GIT1 expression was high in metastatic melanoma tissues. **c** TCGA data revealed the effect of GIT1 expression on the overall survival (OS) of melanoma patients (Kaplan–Meier analysis). **d** TCGA data showed the effect of GIT1 expression on the disease specific survival (DSS) of melanoma patients. **e** GIT1 mRNA expression was observably upregulated in melanoma tissues compared with normal skin tissues. **f** GIT1 protein expression was increased in melanoma tissues. *p < 0.01

TCGA data showed that the GIT1 expression was markedly upregulated in melanoma tissues compared with normal skin tissues (Fig. 5a; p < 0.001). The GIT1 expression was significant higher in metastatic melanoma tissues than in tumor and normal skin tissues (Fig. 5b; p < 0.001). The high GIT1 expression was correlated to the overall survival and disease specific survival of melanoma patients (Fig. 5c, d; p < 0.01). The qRT-PCR assay revealed that GIT1 mRNA expression was remarkably upregulated in 75.8% (50/66) of the melanoma tissues (Fig. 5e; p < 0.001). The GIT1 protein expression was observably increased in melanoma tissues (Fig. 5f). Furthermore, the high GIT1 mRNA expression was relevant to melanoma thickness, lymph node metastasis and initial stage. But the high expression was not associated with age, gender and anatomic site (Additional file 4: Table S6).

### GIT1 promotes melanoma cell proliferation in vitro

**Fig. 6:**
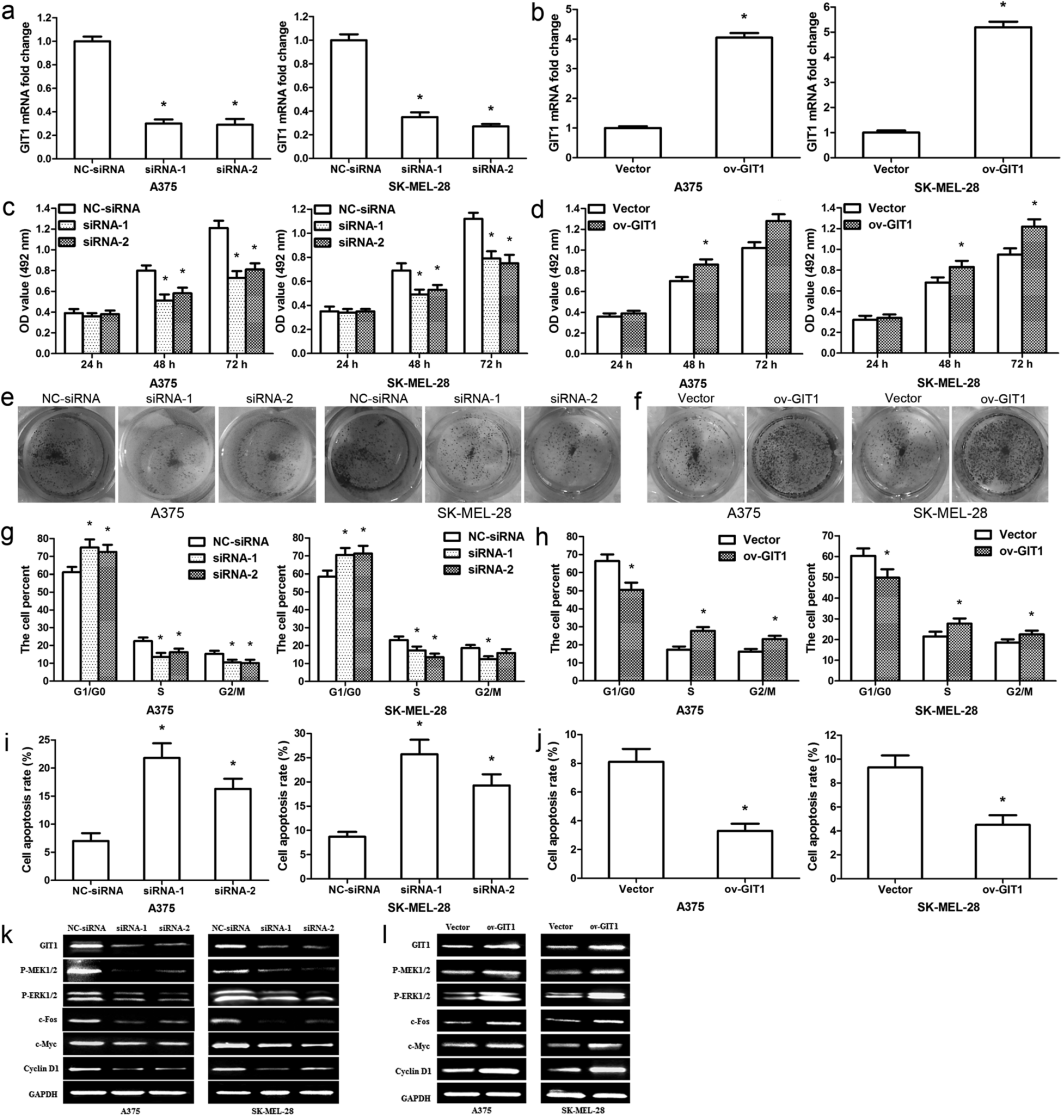
GIT1 accelerates melanoma cell growth and inhibits apoptosis in vitro. **a** GIT1 mRNA expression was examined in A375/SK-MEL-28 cells after silencing GIT1. **b** GIT1 mRNA expression was measured after overexpressing GIT1. **c** MTT results showed melanoma cell activity after silencing GIT1. **d** MTT results revealed melanoma cell activity after overexpressing GIT1. **e** Cell colonies were measured 11 days after silencing GIT1. **f** Cell colonies were examined 11 days after overexpressing GIT1. **g** Cell cycle was analyzed. The histograms showed the proportion of G1/G0, S and G2/M phase cells after t silencing GIT1. **h** The histograms showed the percentage of G1/G0, S and G2/M phase cells after overexpressing GIT1. **i** The data represented the percentage of apoptosis after silencing GIT1. **j** The data revealed the ratios of apoptosis after overexpressing GIT1. **k** GIT1-MEK1/2-ERK1/2 signalling pathways were measured after silencing GIT1. **l** GIT1-MEK1/2-ERK1/2 signalling pathways were examined after overexpressing GIT1. *p < 0.01, n = 3

To determine the role of GIT1 in melanoma, GIT1 siRNAs and GIT1 overexpression vector were transfected into A375/SK-MEL-28 cells, independently. The results revealed that GIT1 siRNAs observably downregulated GIT1 mRNA level in A375/SK-MEL-28 cells (Fig. 6a; p < 0.01), and GIT1 overexpression vector significantly upregulated GIT1 mRNA level (Fig. 6b; p < 0.01). MTT assay showed that silencing GIT1 remarkably suppressed melanoma cell multiplication at 48 and 72 h after transfection (Fig. 6c; p < 0.01), while overexpressing GIT1 significantly facilitated melanoma cell growth (Fig. 6d; p < 0.01). The similar result was observed for colony formation assay (Fig. 6e, f). Cell cycle analysis showed that silencing GIT1 significantly increased the percentage of G1/G0 phase cells and decreased the proportion of S and G2/M phase cells (Fig. 6g; p < 0.01); while overexpressing GIT1 evidently reduced the ratio of G0/G1 phase cells and accelerate the percentage of S and G2/M phase cells (Fig. 6 h; p < 0.01). Furthermore, apoptosis analysis verified that silencing GIT1 markedly induced apoptosis, whereas overexpressing GIT1 remarkably inhibited apoptosis (Fig. 6i, j; p < 0.01). Western blot further measured the downstream signal molecules involved in the promotion of growth by GIT1. The results revealed that silencing GIT1 downregulated GIT1, P-MEK1/2, P-ERK1/2, c-Fos, c-Myc and Cyclin D1 protein expressions, but overexpressing GIT1 upregulated their protein levels (Fig. 6k, l).

### UBTF promotes melanoma cell growth via modulation of MEK1/2-ERK1/2 signalling pathways by enhancing GIT1 transcription

**Fig. 7:**
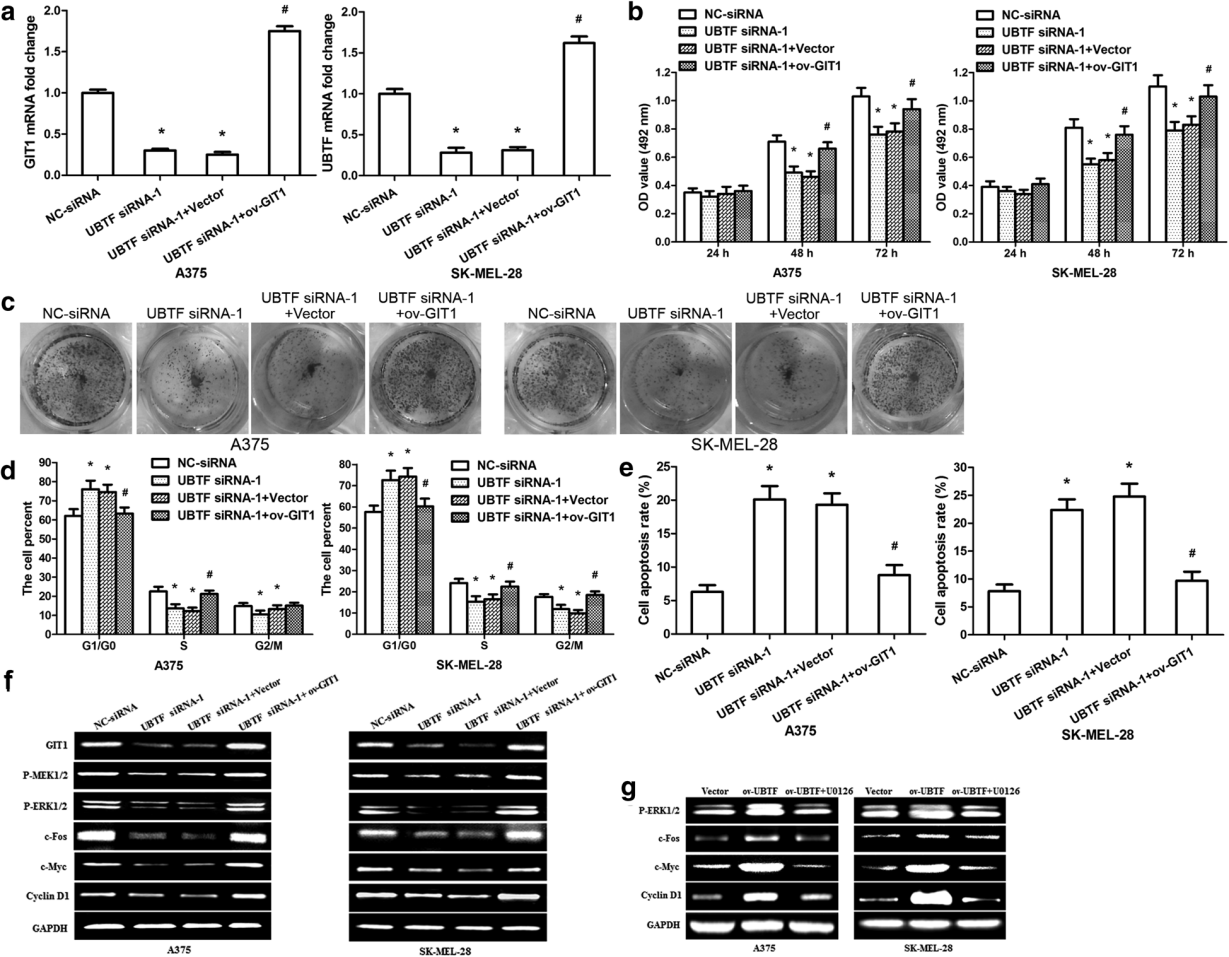
UBTF facilitates melanoma cell growth through modulating GIT1-MEK1/2-ERK1/2 pathways. **a** GIT1 mRNA level was measured in A375/SK-MEL-28 cells after co-transfection with UBTF siRNA-1 and GIT1 overexpression vector. **b** MTT assay revealed melanoma cell multiplication after co-transfection with UBTF siRNA-1 and GIT1 overexpression vector. **c** Cell colonies were detected 11 days after co-transfection. **d** Cell cycle was examined after co-transfection. **e** Apoptosis was analyzed after co-transfection. **f** GIT1-MEK1/2-ERK1/2 signalling pathways were measured after co-transfection. **g** ERK1/2 signalling pathways were detected after co-treatment with UBTF overexpression vector and U0126. *p < 0.01, as compared with NC-siRNA group; ^#^p < 0.01, as compared with UBTF siRNA-1 + vector group; n = 3

To further confirm that UBTF facilitates melanoma cell growth by increasing GIT1 transcription, UBTF siRNA-1 and GIT1 overexpression vector were co-transfected into A375/SK-MEL-28 cells. Silencing UBTF resulted in downregulation of GIT1 mRNA expression, and overexpressing GIT1 reversed the effect of UBTF siRNA-1 on GIT1 mRNA expression (Fig. 7a; p < 0.01). Cell viability assay showed that silencing UBTF inhibited melanoma cell multiplication, overexpressing GIT1 rescued the effect of UBTF siRNA-1 on cell growth (Fig. 7b; p < 0.01). Colony formation assays also revealed the similar results (Fig. 7c). Cell cycle results revealed that silencing UBTF resulted in a prominent increase of G1 phase cells and and a evidently reduction of S and G2/M phase cells in A375/SK-MEL-28 cells, the cells co-transfected with UBTF siRNA-1 and GIT1 overexpression vector were able to re-enter the S and G2/M phases (Fig. 7d; p < 0.01). Evaluation of apoptotic status showed that silencing UBTF remarkably increased apoptosis in A375/SK-MEL-28 cells, while co-transfection with UBTF siRNA-1 and GIT1 overexpression vector reversed the effect of UBTF siRNA-1 on apoptosis (Fig. 7e; p < 0.01). It was reported that GIT1 regulates MEK1/2-ERK1/2 signalling pathways. We detected key molecules in the signalling pathways and discovered that silencing UBTF decreased GIT1, P-MEK1/2, P-ERK1/2, c-Fos, c-Myc and Cyclin D1 protein expressions, co-transfection with UBTF siRNA-1 and GIT1 overexpression vector rescued their protein levels (Fig. 7f). Next, MEK1/2 inhibitor was used to analyze the function of MEK1/2-ERK1/2 signalling pathways in melanoma progression. A375/SK-MEL-28 cells were intervened with UBTF overexpression vector and MEK1/2 inhibitor (U0126). MTT and colony formation assays revealed that U0126 counteract the effect of overexpressing UBTF on cell growth (Additional file 3: Fig. S3a, b; p < 0.01). UBTF overexpression promoted cell cycle progression, while U0126 neutralized the influence of UBTF overexpression on cell cycle (Additional file 3: Fig. S3c; p < 0.01). Furthermore, UBTF overexpression observably suppressed apoptosis, but U0126 reversed the function of UBTF overexpression (Additional file 3: Fig. S3d; p < 0.01). The signalling pathways detection showed that UBTF overexpression promoted P-ERK1/2, c-Fos, c-Myc and Cyclin D1 protein expressions, while U0126 neutralize the effect (Fig. 7g). Our data confirmed that UBTF facilitates melanoma cell proliferation and cell cycle progression by promoting GIT1 transcription, thereby activating MEK1/2-ERK1/2 signalling pathways.

## Discussion

UBTF is an HMGB-box DNA binding protein and a necessary Pol I/Pol II basal transcription factor [15, 16]. UBTF can modulate binding of the pre-initiation factor SL1/TIF1B and assemble pre-initiation complex on ribosomal DNA promoter region. Moreover, UBTF displaces histone chromatin on transcribed regions of ribosomal DNA by forming a nucleosome like structure, and modulates RNA polymerase I transcription elongation responding growth factor signalling [17–19]. Some studies have reported that UBTF, as a regulator of gene expression, involves in carcinogenesis and progression of a few cancers, such as colorectal cancer, breast cancer, lung cancer, cervical cancer and liver cancer [12, 20–22]. It is found that S6K-mediated UBTF promotes colorectal cancer cell proliferation by facilitating the transcription ribosomal DNA [12]. Peng et al. reported that UBTF facilitated osteosarcoma cell proliferation through binding the ribosomal DNA promoter and interacting with 1A6/DRIM at the promoter [23]. Another study demonstrates that loss of UBTF suppresses mouse embryonic fibroblasts proliferation and DNA replication, resulting in cell cycle arrest [24]. Nevertheless, the function of UBTF in other cancers is still unknown. This study revealed that UBTF was upregulated in melanoma and was related to clinicopathologic characteristics. UBTF facilitated melanoma cell proliferation in vitro and in vivo, and the promotion was achieved through promoting cell cycle G1-S phase transition. Furthermore, loss of UBTF also induced nuclear disruption and a rapid and highly penetrant apoptosis, particularly in cells subjected to oncogenic stress [24]. Our results showed that UBTF could restrain melanoma cell apoptosis. The findings indicate that UBTF play a crucial function in melanoma.

To explore the possible molecular mechanism of UBTF regulation in melanoma, we fulfilled bioinformatics predicting, ChIP-qRT-PCR and reporter gene assay, through which we ascertained GIT1 as UBTF targeting gene. It was demonstrated that UBTF promoted GIT1 expression in melanoma cells through binding to the promoter region of GIT1. GIT1 is a multifunctional scaffold protein and has multiple domains, containing paxillin-binding domains, three ankyrin repeat, Spa2 homology, ARFGAP and coiled-coil, so it can interact with various signalling molecules [25, 26]. GIT1 is involved in many biological processes, including cell migration, cell adhesion, cell growth, apoptosis, turnover of focal adhesions, angiogenesis, spine morphogenesis, lamellipodia formation, and synapse formation [27–30]. It is reported that GIT1 expression is upregulated and is a biomarker for tumor prognosis in lung, oral, gastric, breast, colorectal, esophagus and prostate cancers [31, 32]. Recently, it is found that GIT1 plays a crucial function in regulating tumorigenesis and progression. For example, GIT1 promotes the migration of lung cancer cells through activating the activity of Rac1/Cdc42 [33]. Silencing GIT1 inhibits the metastasis of oral squamous cell carcinoma cells [31]. GIT1 can promote gastric cancer cell proliferation and metastasis [34]. In the present study, we found that GIT1 expression was upregulated in melanoma. GIT1 promoted melanoma cell proliferation and suppressed apoptosis in vitro. The rescue studies demonstrated that UBTF facilitated melanoma cell proliferation, cell cycle progression, and inhibited apoptosis through promoting GIT1 transcription.

**Fig. 8:**
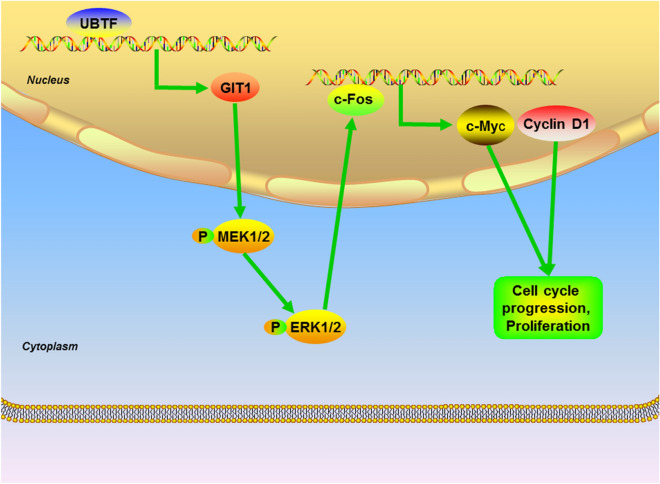
Proposed model for the roles of UBTF on melanoma proliferation. UBTF promotes melanoma cell multiplication by binding to the GIT1 promoter region and accelerating GIT1 transcription, thereby further activating MEK1/2-ERK1/2 signalling pathways

It has been found that GIT1 can regulate multiple signalling pathways refered to tumorigenesis and progression, for example MEK1/2-ERK1/2, Notch, Rac1/Cdc42, Rho, NF-κB, integrin-β1 and P21-activated kinase signalling pathways [35–38]. MEK1/2, as a pivotal member of the Ras-Raf-MEK-ERK MAPK signalling pathway, is activated by GIT1, which in turn activates ERK1/2 [39]. ERK signalling pathway plays a significant role in carcinogenesis, and is frequently disordered in many cancers [40]. Previous studies have shown that ERK signalling pathway facilitates tumorigenesis and progression of melanoma [41]. In this study, our results revealed that UBTF could activate MEK1/2-ERK1/2 signalling pathways through facilitating GIT1 transcription. MEK inhibitor U0126 reversed the effects of UBTF overexpression. These findings suggest UBTF promotes melanoma cell proliferation through promoting GIT1 transcription, thereby activating MEK1/2-ERK1/2 signalling pathways (Fig. 8).

## Conclusions

In summary, this study demonstrates that UBTF functions as an oncogene in melanoma. We found that UBTF is overexpressed and related to the clinicopathologic characteristics of melanoma patients. UBTF promotes melanoma cell proliferation and cell cycle progression by facilitating GIT1 transcription, thereby activating MEK1/2-ERK1/2 signalling pathways. This study suggests that UBTF plays a crucial function in melanoma and may be a potential therapeutic target for the treatment of this disease.

## Supplementary Information

**Additional file 1: Figure S1.** A375 cell proliferation is suppressed aftertransfection with UBTF shRNA in vitro. **a** UBTFmRNA expressionwas measured by qRT-PCR after transfection. **b **UBTF protein expression was detected by Western blot. **c **MTT assay revealed that UBTF shRNA restrained A375 cell proliferation at48 h, and 72 h after transfection. **d **Colonyformation assay. **e** Flow cytometryanalysis revealed the percentages of cells in G1/G0, S, and G2/M phases. **f** The histograms showed the percentagesof apoptosis cells. *p < 0.01, n = 3.

**Additional file 2: Figure S2. **TCGAdata shows that UBTF expression is positivelycorrelated with GIT1 expression.** a **Thecorrelation between UBTF expressionand GIT1 expression in normal tissues.** b**Thecorrelation between UBTFexpression and GIT1 expression in all tissues.

**Additional file 3: FigureS3.** MEK1/2 inhibitor(U0126) reverses the effect of UBTF overexpression in melanoma cell. **a** MTT assay revealed cell viabilityafter co-treatment with UBTF overexpression vector and U0126. **b** Colony formation assay showed cellproliferation after co-treatment with UBTF overexpression vector and U0126. **c** Cell cycle was measured after co-treatment.**d** Apoptosis was detected after co-treatment.*p < 0.01, as compared with vector group; ^#^p < 0.01, as compared withUBTF overexpression vector group; n = 3.

**Additional file 4: Table S1.** Sequences of siRNA. **Table S2.** Sequencesof recombinant plasmids. **Table S3. **Primer sequence used for qRT-PCR or ChIP-qRT-PCR.**Table S4.** Information on antibodies used for the correlationanalysis. **TableS5.** Association between UBTF mRNA expression and clinical pathological featuresof melanoma (n = 66). **Table S6.** Relationshipbetween GIT1 mRNA expression and clinical pathological characteristics ofmelanoma (n = 66).

## Data Availability

The datasets used in this study are available from the corresponding author upon reasonable request.

## Abbreviations

ChIP: Chromatin immunoprecipitation
DMEM: Dulbecco’s Modified Eagle’s Medium
GITI: G-protein-coupled receptor kinase-interacting protein 1
PBS: Phosphate-buffered saline
qRT-PCR: Quantitative real time PCR
siRNAs: Small interfering RNAs
UBTF: Upstream binding transcription factor
