# Supplementary figures and images for: UBTF facilitates melanoma progression via modulating MEK1/2-ERK1/2 signalling pathways by promoting GIT1 transcription
Source: Cancer Cell Int. 2021 Oct 18;21:543. doi: 10.1186/s12935-021-02237-8 (PMC8522148; doi:10.1186/s12935-021-02237-8)

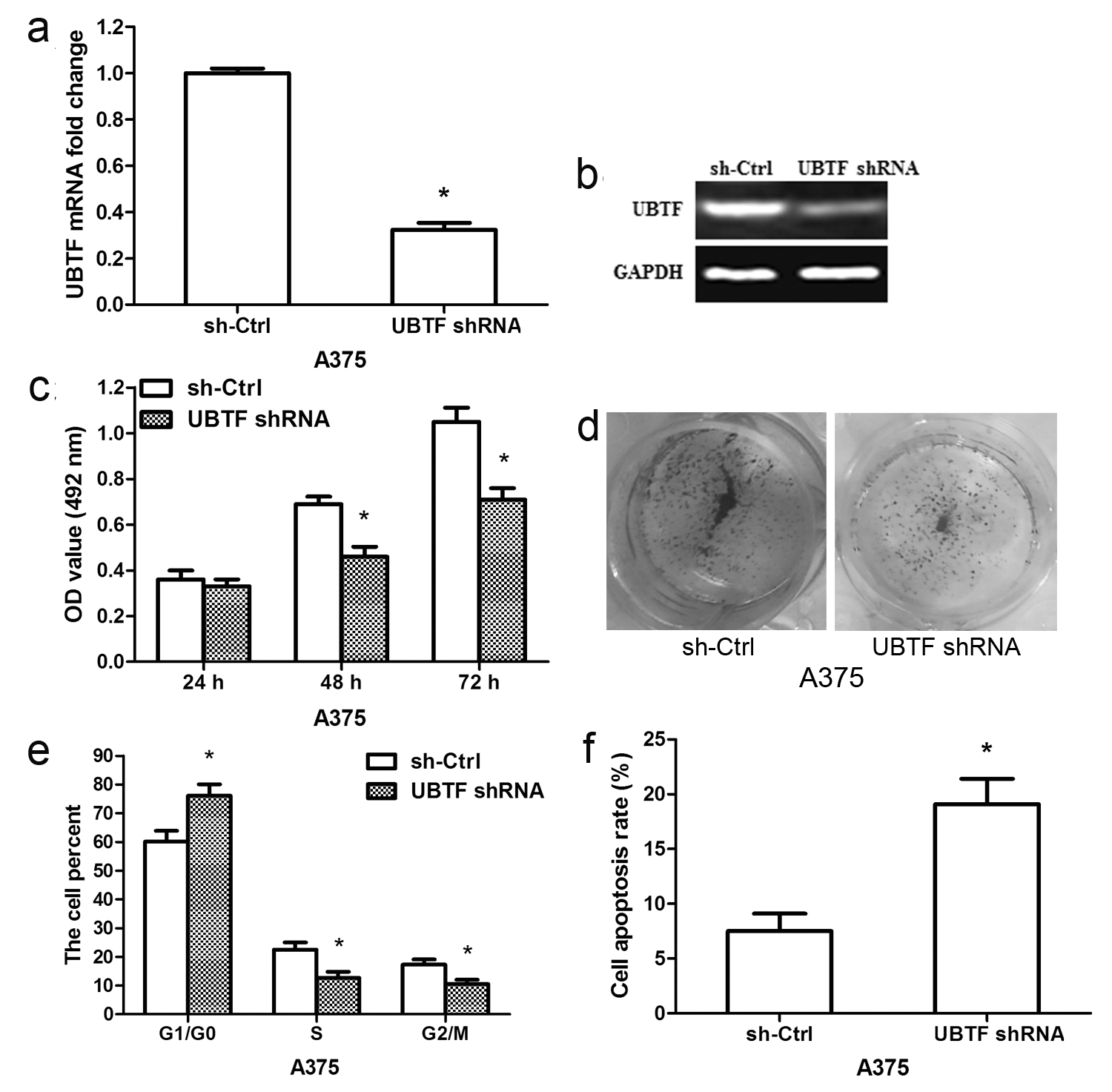

Supplement: Supplementary file 1 — Additional file 1: Figure S1. A375 cell proliferation is suppressed aftertransfection with UBTF shRNA in vitro. a UBTFmRNA expressionwas measured by qRT-PCR after transfection. b UBTF protein expression was detected by Western blot. c MTT assay revealed that UBTF shRNA restrained A375 cell proliferation at48 h, and 72 h after transfection. d Colonyformation assay. e Flow cytometryanalysis revealed the percentages of cells in G1/G0, S, and G2/M phases. f The histograms showed the percentagesof apoptosis cells. *p < 0.01, n = 3. [file 12935_2021_2237_MOESM1_ESM.tif]

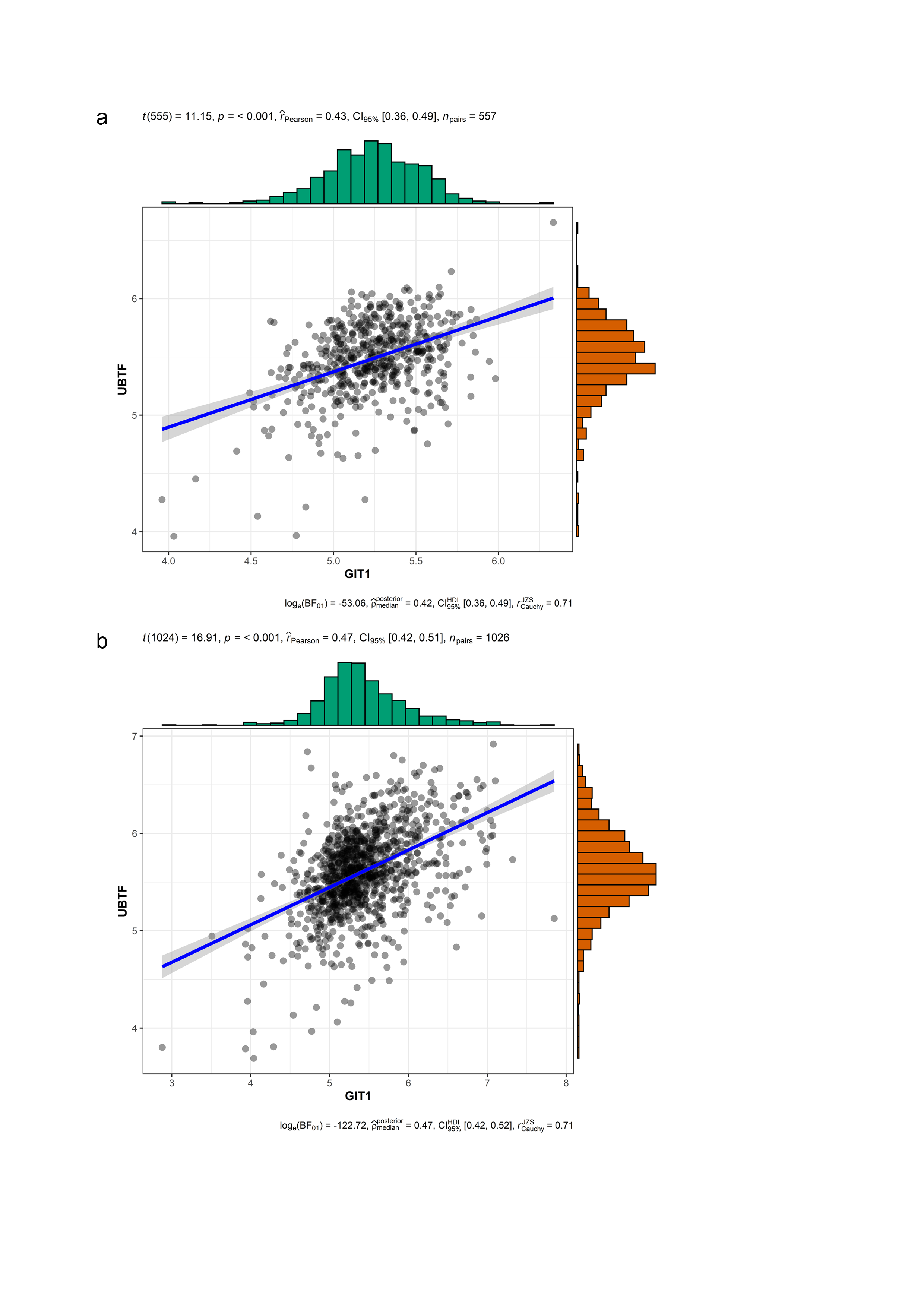

Supplement: Supplementary file 2 — Additional file 2: Figure S2. TCGAdata shows that UBTF expression is positivelycorrelated with GIT1 expression. a Thecorrelation between UBTF expressionand GIT1 expression in normal tissues. bThecorrelation between UBTFexpression and GIT1 expression in all tissues. [file 12935_2021_2237_MOESM2_ESM.tif]

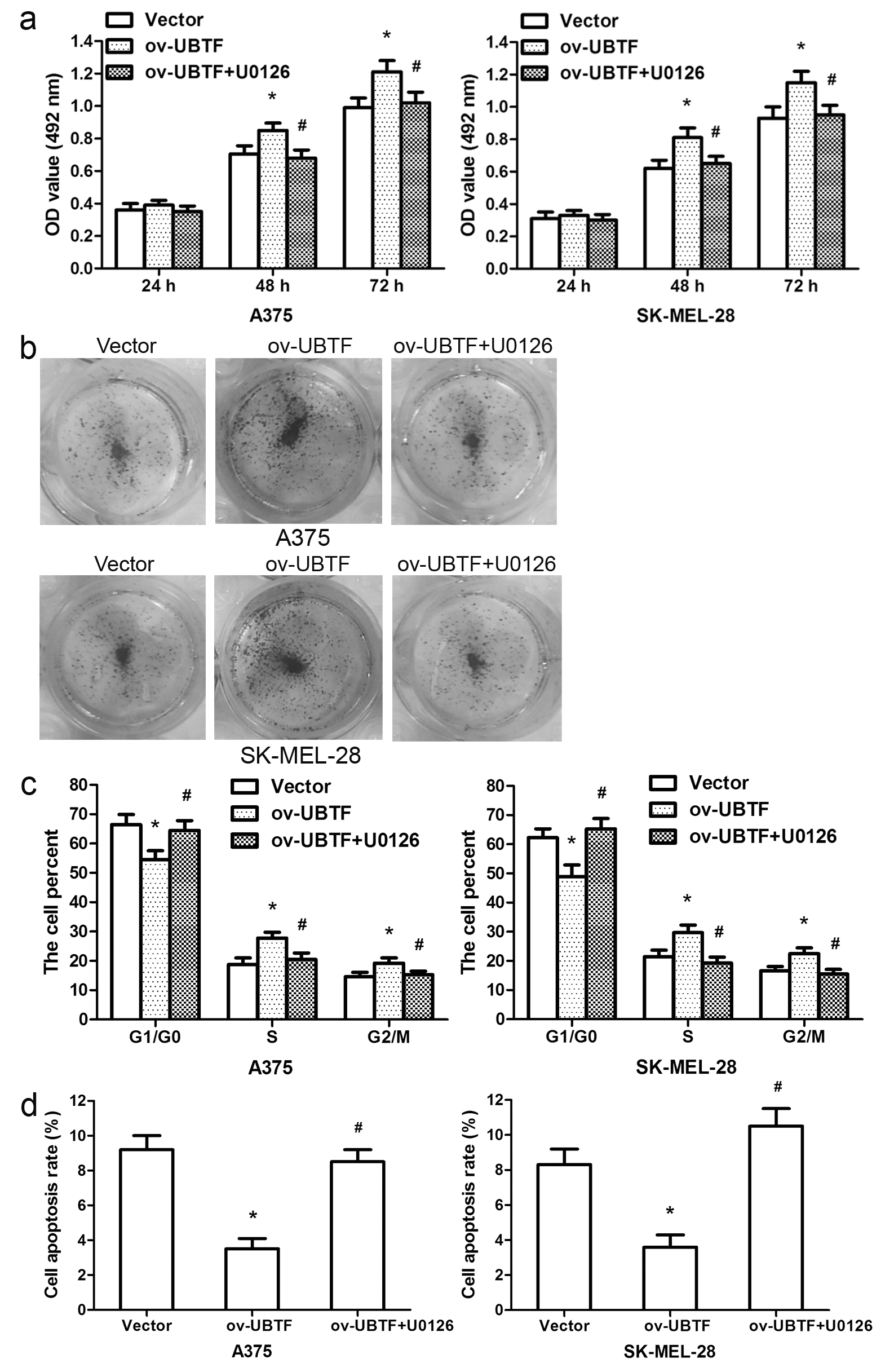

Supplement: Supplementary file 3 — Additional file 3: FigureS3. MEK1/2 inhibitor(U0126) reverses the effect of UBTF overexpression in melanoma cell. a MTT assay revealed cell viabilityafter co-treatment with UBTF overexpression vector and U0126. b Colony formation assay showed cellproliferation after co-treatment with UBTF overexpression vector and U0126. c Cell cycle was measured after co-treatment.d Apoptosis was detected after co-treatment.*p < 0.01, as compared with vector group; #p < 0.01, as compared withUBTF overexpression vector group; n = 3. [file 12935_2021_2237_MOESM3_ESM.tif]
