# Supplementary material for: UBTF facilitates melanoma progression via modulating MEK1/2-ERK1/2 signalling pathways by promoting GIT1 transcription
Source: Cancer Cell Int. 2021 Oct 18;21:543. doi: 10.1186/s12935-021-02237-8 (PMC8522148; doi:10.1186/s12935-021-02237-8)
Supplement: Supplementary file 4 — Additional file 4: Table S1. Sequences of siRNA. Table S2. Sequencesof recombinant plasmids. Table S3. Primer sequence used for qRT-PCR or ChIP-qRT-PCR.Table S4. Information on antibodies used for the correlationanalysis. TableS5. Association between UBTF mRNA expression and clinical pathological featuresof melanoma (n = 66). Table S6. Relationshipbetween GIT1 mRNA expression and clinical pathological characteristics ofmelanoma (n = 66). [file 12935_2021_2237_MOESM4_ESM.docx]

**Table S1** Sequences of siRNA

| Name | Sequence | |
| --- | --- | --- |
| negative siRNA (NC-siRNA) sense | 5′-UUCUCCGAACGUGUCACGUTT-3′ |  |
| negative siRNA (NC-siRNA) antisense | 5′- ACGUGACACGUUCGGAGAATT-3′ | |
| UBTF siRNA-1 sense | 5′- GGAGAAACUGAUGUGGAUUTT-3′ | |
| UBTF siRNA-1 antisense | 5′- AAUCCACAUCAGUUUCUCCTT-3′ | |
| UBTF siRNA-2 sense | 5'- CCUGGGCUUCAAGAAAUGUTT-3' | |
| UBTF siRNA-2 antisense | 5'- ACAUUUCUUGAAGCCCUGGTT-3' | |
| GIT1 siRNA-1 sense | 5'- GUACCAGAUGCUGGCAUUUTT-3' | |
| GIT1 siRNA-1 antisense | 5'- AAAUGCCAGCAUCUGGUACTT-3' | |
| GIT1 siRNA-2 sense | 5'- CACUGGAAAGUCUGUUUUUTT-3' | |
| GIT1 siRNA-2 antisense | 5'- AAAAACAGACUUUCCAGUGTT-3' | |

**Table S2** Sequences of recombinant plasmids

| Name | Sequence |
| --- | --- |
| Negative control  UBTF shRNA | 5′-AAAAGAGGCTTGCACAGTGCATTCAAGACGTGCACTGTGCAAGCCTCTTTT-3′  5'-TGGAGAAACTGATGTGGATTTTCTCGAGAATCCACATCAGTTTCTCCTTTTTTC-3' |

**Table S3** Primer sequence used for qRT-PCR or ChIP-qRT-PCR

| Gene | Sequence |
| --- | --- |
| UBTF-F | 5'-AGGCGGAAAGGCAGGCAAGATGGTG-3' |
| UBTF-R | 5'-CAAGGAAGGCTGGAGGGATGTGTAA-3' |
| GIT1-F | 5'-CTAGCTAGCGCGTCGCCGCTGAGGA-3' |
| GIT1-R | 5'-GGAATTCGGGGCGCATGTACGGA-3' |
| GAPDH-F | 5'-GCCGTATCGCTCAGACAC-3' |
| GAPDH-R | 5'-GCCTAATACGACCAAATCC-3' |
| GIT1 (ChIP)-Primer 1-F  GIT1 (ChIP)-Primer 1-R  GIT1 (ChIP)-Primer 2-F  GIT1 (ChIP)-Primer 2-R  GIT1 (ChIP)-Primer 3-F  GIT1 (ChIP)-Primer 3-R  GIT1 (ChIP)-Primer 4-F  GIT1 (ChIP)-Primer 4-R  GIT1 (ChIP)-Primer 5-F  GIT1 (ChIP)-Primer 5-R  GIT1 (ChIP)-Primer 6-F  GIT1 (ChIP)-Primer 6-R  GIT1 (ChIP)-Primer 7-F  GIT1 (ChIP)-Primer 7-R  GAPDH (ChIP)-F  GAPDH (ChIP)-R | 5'-GGGGCCTGGCAAGAGCGCTGTGCGG-3'  5'-GGCGCAGCTCCGCAGTGGCCGAGGC-3'  5'-GGGCTGCGGCCTCCAGGCTGGGCCC-3'  5'-CACGGCCTCCAGGGAGCATGGGCAC-3'  5'-CCTTGGCGGGAGGGCCGGCCTGGGG-3'  5'-CTCAGACCGAGGAGCGGGAGGCGGC-3'  5'-ACTAGCGAGGAAGGACGTTCCCGGC-3'  5'-TGGCGCTTCCGTCGGATACAGAGAT-3'  5'-CTGGCTGCTCCTCCACCTCCATCTC-3'  5'-CGCCCCCACCTCCCAAGCCCTGACG-3'  5'-CTGGGGTGGCAGGAGGGCCGTGGCG-3'  5'-AGCAGCAGGTGCAAGACGCGGGGGT-3'  5'-TTTATGTCATCTCAGTGCCACCCCC-3'  5'-TCTGCCCGGCCCTCTGCTCCTTGCC-3'  5'-GTGGCAAAGTGGAGATTGTT-3'  5'-CTCGCTCCTGGAAGATGG-3' |

**Table S4** Information on antibodies used for the correlation analysis

| Antibody | WB | Specificity | | Company | |
| --- | --- | --- | --- | --- | --- |
| UBTF  GIT1  Phospho-MEK1/2  Phospho-ERK1/2  c-Myc  c-Fos  Cyclin D1 (2978)  GAPDH  UBTF ChIP Grade  IgG ChIP Grade | 1:1000  1:1000  1:1000  1:1000  1:1000  1:1000  1:1000  1:3000  -  - | Rabbit polyclonal  Mouse Monoclonal  Rabbit Monoclonal  Rabbit Monoclonal  Rabbit Monoclonal  Rabbit Monoclonal  Rabbit Monoclonal  Mouse Monoclonal  Rabbit polyclonal  Rabbit polyclonal | Santa Cruz Biotechnology  Santa Cruz Biotechnology  Cell Signaling Technology  Cell Signaling Technology  Cell Signaling Technology  Cell Signaling Technology  Cell Signaling Technology  Santa Cruz Biotechnology  Abcam  Abcam | |  |

**Table S5** Association between UBTF mRNA expression and clinical pathological features of melanoma (n = 66).

| Characteristics | Number of cases | | | UBTF mRNA expression | | | p-value |
| --- | --- | --- | --- | --- | --- | --- | --- |
|  |  |  |  | High (n = 53) | | Low (n = 13) |  |
| Age | |  |  | |  | | 0.836 |
| ≥60 years | 35 | | 28 | | 7 | |  |
| <60 years | 31 | | 25 | | 6 | |  |
| Gender |  | |  | |  | | 0.725 |
| Male | 32 | | 27 | | 5 | |  |
| Female | 34 | | 26 | | 8 | |  |
| Anatomic site |  | |  | |  | | 0.588 |
| Acra | 24 | | 19 | | 5 | |  |
| Trunk  Other | 28  14 | | 22  12 | | 6  2 | |  |
| Thickness  <2 mm  ≥2 mm  Lymph node metastasis Yes  No | 40  26  21  45 | | 29  24  19  34 | | 11  2  2  11 | | 0.006*    0.013* |
| Initial Stage |  | |  | |  | | 0.003* |
| I+II | 30 | | 20 | | 10 | |  |
| III+IV | 36 | | 33 | | 3 | |  |

*p < 0.05

**Table S6** Relationship between GIT1 mRNA expression and clinical pathological characteristics of melanoma (n = 66).

| Characteristics | Number of cases | | | GIT1 mRNA expression | | | p-value |
| --- | --- | --- | --- | --- | --- | --- | --- |
|  |  |  |  | High (n = 50) | | Low (n = 16) |  |
| Age | |  |  | |  | | 0.855 |
| ≥60 years | 35 | | 26 | | 9 | |  |
| <60 years | 31 | | 24 | | 7 | |  |
| Gender |  | |  | |  | | 0.887 |
| Male | 32 | | 24 | | 8 | |  |
| Female | 34 | | 26 | | 8 | |  |
| Anatomic site |  | |  | |  | | 0.452 |
| Acra | 24 | | 18 | | 6 | |  |
| Trunk  Other | 28  14 | | 21  11 | | 7  3 | |  |
| Thickness  <2 mm  ≥2 mm  Lymph node metastasis Yes  No | 40  26  21  45 | | 28  22  18  32 | | 12  4  3  13 | | 0.008*    0.016* |
| Initial Stage |  | |  | |  | | 0.005* |
| I+II | 30 | | 19 | | 11 | |  |
| III+IV | 36 | | 31 | | 5 | |  |

*p < 0.05
